# Economic cost–utility analysis of stage-directed gastric cancer treatment

**DOI:** 10.1093/bjsopen/zrab129

**Published:** 2022-01-12

**Authors:** Arfon G Powell, Jennifer R Wheat, Catherine Eley, David Robinson, Stuart A Roberts, Wyn Lewis

**Affiliations:** 1 Division of Cancer and Genetics, Cardiff University School of Medicine, Cardiff, UK; 2 Department of General Surgery, University Hospital of Wales, Cardiff, UK; 3 Department of Radiology, University Hospital of Wales, Cardiff, UK

## Abstract

**Background:**

Gastric cancer (GC) treatment levies substantial financial burden on health services. Potentially curative surgery with or without chemotherapy is offered to patients with locoregional disease. This study aimed to examine treatment costs related to life-years gained in patients having potentially curative treatment (gastrectomy) and those receiving best supportive care (BSC).

**Methods:**

Some 398 consecutive patients with GC were classified according to treatment modality (116 BSC, 282 gastrectomy). Cost calculations for 1 year’s treatment from referral were made according to network diagnostic, staging and treatment algorithms. Primary outcome was overall survival (OS).

**Results:**

GC median survival after BSC was 8 months, costing €5413, compared with gastrectomy median survival of 34 months, costing €22 753 for 1 year’s treatment: cost per life-year gained €9319. Cost incurred for stage I GC was €22 434, stage II €23 498, stage III €22 445, and stage IV €22 032. Based on these values, the cost per quality adjusted life-year (QALY) for BSC for stage I GC was –€8335 stage II –€8952, stage III –€11 317, and stage IV –€25 669.

**Conclusion:**

Potentially curative treatment that included gastrectomy improved OS four-fold compared with BSC and was cost-effective at national thresholds of readiness to pay per QALY.

## Teaser

Cost incurred for stage I gastric cancer (GC) was €22 434 €23 498, stage III €22 445, and stage IV €22 032. Based on these values, the cost per quality adjusted life-year (QALY) for BSC for stage I GC was –€8335 stage II –€8952, stage III –€11 317, and stage IV –€25 669. Potentially curative gastrectomy for GC improved OS four-fold compared with BSC and is cost-effective at national thresholds of readiness to pay per QALY.

## Introduction

Gastric cancer treatment is directed by the perceived stage at diagnosis, derived from the UICC TNM system^1^^,^^2^. Prognosis worsens with increasing stage so differential treatment quotients can be inferred.

Potentially curative gastric cancer treatment involves surgical resection, but is only feasible in some 25 per cent of patients^3–5^. A variety of palliative treatments can be used including endoscopic stenting, chemotherapy or radiotherapy, as well as no treatment for those who choose this option or who are deemed unfit for any intervention. These patients who are treated with no intention to achieve cure are described as receiving best supportive care (BSC)^1^^,^^6^.

Clinical effectiveness can be estimated in terms of patient quality adjusted life-years (QALYs)^7^, that considers a matrix of treatment cost, quality of life (QoL) and overall survival. Treatment costs are estimated relative to the healthcare system rather than society^8^, and QoL is measured with validated questionnaires^9^^,^^10^, that can be transformed into proportions termed utility values (UV). In the UK, the National Institute for Health and Care Excellence (NICE) uses a cost per QALY factor to determine whether a given treatment can be endorsed within the National Health Service (NHS). This results in defined scales regarding treatment: thresholds of €23 233 (£20 000) per QALY equating to recommended, €34 850 (£30 000) likely recommendation, with titration to €58 083 (£50 000) for palliative end-of-life treatment^7^^,^^11^^,^^12^.

The aim of this study was to estimate the cost–utility of treatment related to the stage of gastric cancer compared with BSC. Primary healthcare outcomes were QALYs gained related to treatment and economic outcome was incremental cost-effectiveness ratio (ICER).

## Methods

###  

Consecutive patients diagnosed with gastric adenocarcinoma between 2006 and 2018 were included. Those undergoing curative endoscopic resection were not included in the analysis. All patients were managed by a regional cancer network, which includes surgeons, oncologists, radiologists and pathologists with a subspecialty interest in gastric cancer^4^. Diagnosis was made by oesophagogastroduodenoscopy and biopsy, or by CT alone in those patients considered not suitable for further treatment, due to co-morbidity. Radiological staging consisted of CT thorax, abdomen and pelvis. Patients with cancer arising from the gastro-oesophageal junction also underwent CT-PET and endoscopic ultrasonography (EUS) according to staging protocols published previously^13^. Staging laparoscopy was performed selectively in patients deemed to have potentially curable disease. Staging was recorded according to Union for International Cancer Control TNM classification, 7th edition, for gastric adenocarcinoma^2^^,^^14^. Patients deemed on clinical evaluation to be at high risk of postoperative morbidity underwent cardiopulmonary exercise testing (CPET) as reported previously^15^.

### Treatment options

#### Treatment with non-curative intent

Patients with metastatic disease, inoperable locally advanced disease, or co-morbidity precluding treatment with curative intent received BSC. BSC included therapeutic endoscopy, single fraction palliative radiotherapy for symptomatic anaemia and palliative chemotherapy for patients with appropriate performance status, as well as no treatment.

#### Treatment with curative intent

The surgical treatment algorithm of the SE Wales Upper Gastrointestinal Cancer Network’s surgical team has been reported previously^4^^,^^16^. Selective use of neoadjuvant chemotherapy was adopted following publication of the MAGIC trial^17^. Patients with minimal co-morbidity who were deemed to have relatively advanced disease considered likely to benefit from downstaging prior to surgery received chemotherapy, administered for three or four cycles before and after surgery. Each cycle consisted of epirubicin (50 mg/m^2^) by intravenous bolus, cisplatin (60 mg/m^2^) as a 4-hour infusion on day 1 and 5-fluorouracil (200 mg/m^2^/day) daily by a continuous intravenous infusion.

Patients who at the time of surgery had metastatic disease or inoperable locally advanced disease underwent palliative bypass procedures or open and close laparotomy. These patients were analysed on an intention to treat basis and characterized as TNM stage IV.

Ethical approval was sought, but the chair of Cardiff & Value University Health Board ethics committee confirmed that individual patient consent was not required to report clinical outcomes alone, and no formal approval was necessary.

#### Economic analysis

All available management options were analysed and compared with BSC and included single-modality surgery and surgery augmented by perioperative chemotherapy^4^^,^^6^. The patient pathways for these options were classified according to the principle of operational efficiency: using the minimum necessary resources to deliver a particular activity. Management of treatment-related co-morbidity was not included in cost calculations.

Costs to the NHS were calculated, but personal and societal costs were not. Activity-based costs were used preferentially to maximize regional accuracy. CPET, EUS and out-of-region service provision of CT-PET were calculated using activity-based costing. Contemporary NHS England reference costs were used for outpatient appointment and critical care stay costs, where bottom-up costing approaches were not possible^18^. Reference costs used a top-down approach, increasingly used for economic analysis in the UK, and adopted for use in Scotland and Wales^19^. Staff costs were taken from the Personal Social Services Research Unit^20^, using cost per hour, including the capital training costs divided over the total whole time equivalent hours of service. Length of time per patient interaction was obtained from personal communications with the staff involved. Medication costs were taken from the British National Formulary, chemotherapy regimens were costed according to local protocols, based on contemporary trial data^17^. Procurement costs for disposables in theatre, and operating theatre running costs, per minute, were obtained from Information Services Division Scotland^21^. Chemotherapy doses were calculated according to an average male height and weight of 1.75 m and 70 kg, giving a body surface area of 1.85 m^2^, according to the Du Bois formula^22^. Costs were calculated from referral for a year of treatment and follow-up within that year. Costs are presented in euros (EUR), converted from pounds sterling (GBP) using a conversion rate of 1 GBP to 1.18 EUR as of 9 August 2021.

To determine health-related QoL, mapping techniques applied to translate EORTC QLQ-C30 scores into EQ-5D Health Status Utility Values (HSUVs) were retrieved from the literature. There were no separate QoL data in the literature for patients undergoing surgery and perioperative chemotherapy compared with those undergoing surgery alone, and therefore a coefficient of 0.866 was used for both^23^. For patients undergoing BSC, an HSUV of 0.576 was applied^24^.

### Statistical analysis

Overall survival was calculated from the date of diagnosis recorded to the date of death as recorded from the Office of National Statistics feed into Cancer Network Information System Cymru or censoring. Non-parametric statistical methods were used and median values were used for grouped data. Treatment costs were given as mean values. Median overall survival per stage and per treatment was calculated using the Kaplan–Meier estimator. A non-parametric test of independent samples was performed to identify statistically significant differences in survival at a probability level (*P* value) of less than 0.050.

QoL data were derived from the published literature^23^^,^^24^. The HSUVs were on a zero to one scale, where zero represented death and one full health. Mapping techniques were used to convert questionnaire outcomes without HSUVs to scores from which HSUVs could be generated. QALYs were calculated by multiplying the time spent in each HSUV (measured in years) by the utility score. The costs of all treatments were initial outlay costs, and no delayed costs were included, so no discount rate was applicable. All statistical analysis was performed in SPSS^®^ version 25.0 (IBM Corporation, Armonk, New York, USA) with extension R.

## Results

Some 398 consecutive patients diagnosed with gastric adenocarcinoma were studied; 282 treated with curative intent and 116 given BSC. Patients undergoing curative endoscopic resection were not included. Of those receiving curative treatment, 85 (30.1 per cent) received perioperative chemotherapy, with 233 (82.6 per cent) undergoing gastrectomy. There were 49 (17.4 per cent) patients who, because of locally advanced disease, underwent an open and close laparotomy or palliative bypass procedure, and were consequently classified as stage IV. Of those undergoing gastrectomy, 90 patients (38.6 per cent) underwent subtotal and 143 (61.4 per cent) total gastrectomy. The individual cost of each treatment modality and cost breakdown can be found in *Table 1*, and total cost was €6 619 943. Following intraoperative and pathological assessment of patients undergoing surgery, 72 patients (25.5 per cent) were stage I, 69 (24.5 per cent) stage II, 92 (32.6 per cent) stage III and 49 (17.4 per cent) stage IV. During follow-up 71 patients (25.2 per cent) developed recurrence, and 147 (52.1 per cent) died. The median follow-up of survivors was 32 (i.q.r. 15–60) months.

**Table 1:** Relative cost related to treatment for patients diagnosed with gastric cancer

Treatment pathway	Cost for 1 year of treatment (€)	No. of patients	Total cost (€)
Total gastrectomy and perioperative chemotherapy	27 459	55	1 510 264
Subtotal gastrectomy and perioperative chemotherapy	25 928	18	466 702
Total gastrectomy	21 468	88	1 889 168
Subtotal gastrectomy	19 936	72	1 435 420
Open and close laparotomy and perioperative chemotherapy	24 462	12	293 545
Open and close laparotomy	21 244	37	786 046
Best supportive care	5413	116	627 855

Overall median survival for patients receiving BSC was 8 (range 1–18) months with a QALY-adjusted survival of 4.6 months, and cost per QALY of €14 120. Median survival of patients undergoing potentially curative treatment was 33.8 (range 28.2–39.4) months, with an average cost for 1 year’s treatment of €22 753. This resulted in a QALY-adjusted survival of 29.3 months, with cost per QALY of €9319.

On an intention-to-treat basis, median survival for patients receiving perioperative chemotherapy (CS) was 35.4 (95 per cent c.i. 29.8 to 40.9) months, compared with 30.4 (95 per cent c.i. 21.8 to 38.9) months for patients treated with single-modality primary surgery (S). This resulted in a QALY-adjusted survival of 30.7 and 26.3 months for CS and S, respectively. Given the differential cost encountered with performing total gastrectomy *versus* subtotal gastrectomy, a mean cost was calculated based on the proportions of patients receiving the treatment. The cost of performing CS was €26 712 and S €20 866. Cost per QALY for CS was €10 441 compared with €9521 for S.

Open and close laparotomies were performed in 37 patients (18.8 per cent) in the S cohort compared with 12 (14.1 per cent) in the CS cohort (*P* = 0.343). In patients who underwent gastrectomy, the median survival in the CS cohort was 37.5 (95 per cent c.i. 30.2 to 44.7) months compared with 52.8 (95 per cent c.i. 32.8 to 72.8) months in the S cohort. In patients undergoing gastrectomy, the cost of a QALY in the CS cohort was €8666 compared with €4722 in the S cohort. The QALY-adjusted OS was 32.7 months in the CS cohort compared with 45.7 months in the S cohort. The CS cohort had a slightly greater proportion of patients with pTNM stage I/II disease (55.3 *versus* 47.7 per cent, *P* = 0.243).

**Fig. 1:**
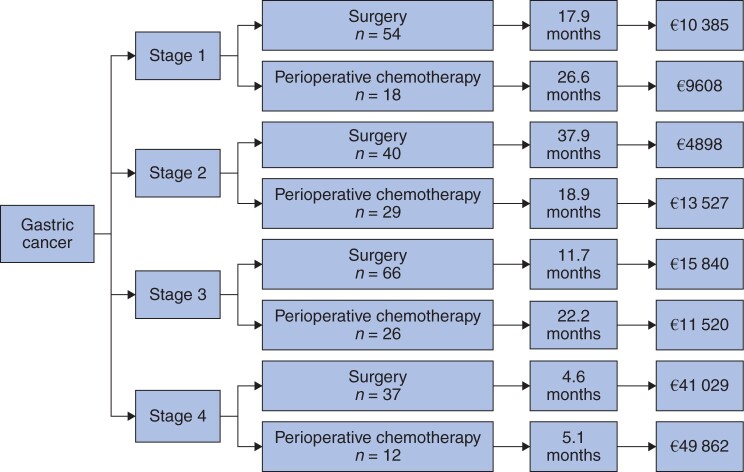
Gastric cancer treatment Treatment stratified by stage and modality: quality adjusted life-year (QALY)-adjusted median survival and cost per QALY

Data relating to QALY-adjusted survival and the cost per QALY, stratified by tumour stage, can be found in *Table 2*. The cost analysis of treating gastric cancer related to TNM stage and treatment modality is shown in *Fig. 1*.

**Table 2:** Cost–utility analysis of treatment of gastric cancer related to disease stage

Tumour stage	Median survival (months)	Average treatment costs (€)	QALY-adjusted survival (months)	Cost per QALY (€)
Stage I	37.3 (4.0–60.0)	22 434	32.3	8335
Stage II	36.4 (11.5–60.0)	23 498	31.5	8952
Stage III	27.5 (19.4–35.7)	22 445	23.8	11 317
Stage IV	11.9 (10.0–13-8)	22 032	10.3	25 669

Values in parentheses are 95 per cent confidence intervals.

**Fig. 2:**
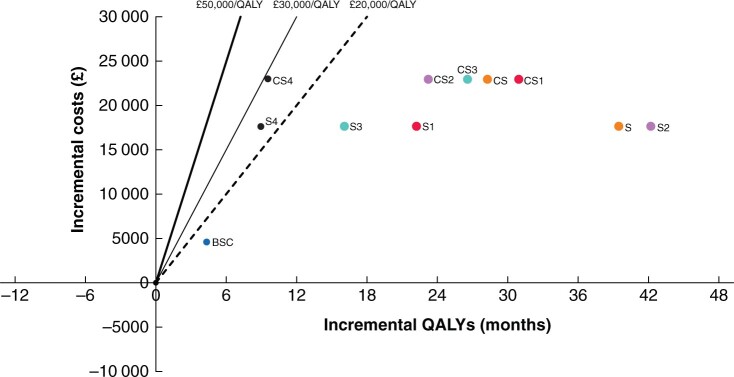
Cost-effectiveness of gastric cancer treatment stratified by stage and treatment modality BSC, best supportive care; S, surgery; CS, perioperative chemotherapy; CS1 and S1, pTNM stage I gastric cancer (GC) treated with perioperative chemotherapy and surgery respectively; CS2 and S2, pTNM stage II GC treated with perioperative chemotherapy and surgery respectively; CS3 and S3, pTNM stage III GC treated with perioperative chemotherapy and surgery respectively; CS4 and S4, pTNM stage IV GC treated with perioperative chemotherapy and surgery respectively (intention to treat).

The relative incremental costs and associated QALYs in OS months for different treatment modalities and tumour stages can be found in *Fig. 2*. BSC had a QALY OS of 4.4 months with an associated cost of €5413. In patients undergoing CS and S, the QALY OS gains were 23.9 and 35.2 months, with an associated increased cost of €21 299 and €15 454 respectively. This equates to an ICER of €7261/QALY for CS and €7759/QALY for S. When further stratified by tumour stage, ICER for TNM stage I was €9608/QALY for CS and €10 385/QALY for S. For stage II, the ICER was €13 527/QALY for CS and €4898/QALY for S. For stage III, the ICER for CS was €11 520/QALY and €15 840/QALY for S. Finally for stage IV, the ICER was €49 862/QALY for CS and €41 029/QALY for S.

## Discussion

Potentially curative surgery for GC improved overall survival four-fold compared with BSC and was cost effective at nationally accepted thresholds of readiness to pay per QALY. The study analysed the full spectrum of disease and range of management. Costs per QALY increased incrementally and proportionately with the stage of gastric cancer so that stage I treatment cost per QALY was a third of that associated with BSC, and stage III treatment cost per QALY less than half that of BSC. Similarly, with regard to ICER-defined cost-effectiveness comparisons, treatment of patients diagnosed with stage I cancer was between four- and five-fold cheaper than treatment of patients diagnosed with stage IV disease. The QALY cost for treating GC with non-curative intent was amplified by poor survival, and offering patients potentially curative treatment, where possible, was evidently more cost-effective.

Economic analyses have been performed in cancers originating from a range of anatomical sites, indicating that stage is associated with a stepwise incremental increase in treatment costs in patients undergoing potentially curative treatments^19^^,^^25^. The poorer survival observed in advanced disease is a major contributor to cost per QALY, as more intensive treatments are required to gain similar survival to that observed in patients with earlier stages of disease. The cost of managing GC in patients with curable disease has been estimated at $42 521 for surgery alone, compared with $156 547^26^^,^^27^ per QALY for human epidermal growth factor receptor type-2 (HER2)-positive metastatic disease treated with trastuzumab chemotherapy.

Treating locally advanced cancer poses a significant economic dilemma, because patients with locally advanced disease or those with lymph node metastasis have the highest risk of disease relapse. Current evidence suggests that only a small proportion of patients, approximately 15–20 per cent, undergoing neoadjuvant therapy and surgery for gastric^28^ and oesophageal cancer^29^^,^^30^ demonstrate significant pathological tumour regression. Improving response to chemotherapy might, therefore, offer the best cost benefit. The additional cost of chemoradiotherapy^31^ in GC treatment regimens has been estimated to be $20 100; resulting in a QALY of 2.25 years compared with 1.72 years after surgery alone—a gain of 0.53 years—equating to an incremental cost-effectiveness ratio of $38 400/QALY. The findings of the present study are in keeping with the above. Patients undergoing perioperative chemotherapy benefited from a 5-month improvement in QALY-adjusted overall survival, when analysed on an intention-to-treat basis. While the present study indicated at least a 10-month QALY-adjusted overall survival benefit associated with perioperative chemotherapy in patients with stages pTNM I and III, this was not the case for stage II cancer, probably reflecting the differential extent of downstaging in these patients. These findings support a precision-medicine approach to managing GC, with chemotherapy tailored to those likely to derive benefit. A cost-effectiveness analysis of the Tratuzumab for Gastric Cancer (ToGA) trial^26^, that compared trastuzumab with platinum-based chemotherapy *versus* chemotherapy alone for metastatic gastric cancer in patients with HER2 amplification or overexpression^32^, showed that patients with tumours demonstrating strong HER2 expression on immunohistochemistry (IHC) derived the most benefit, with an incremental QALY of 0.326 and an ICER of €55 000 compared with €83 000 in IHC2+/FISH+ or IHC3+ tumours and €110 000 in the HER2+ tumours.

There are a number of inherent limitations in the present study. A number of methodological assumptions were made. Operational efficiency (using the minimum necessary resource to deliver a particular activity, in essence the cost of the treatment pathway, did not include the added costs of co-morbidity, and deviations from the pathways, such as repeat MDT discussions, anticoagulant therapy, or repeat visits to complete CT staging). Costs increase markedly if complications occur. Costs were determined by intention to treat, and therefore if a patient’s treatment deviated part way through, costs were incurred but survival influenced differently, such as disease progression during neoadjuvant chemotherapy that meant surgery was no longer appropriate or finding inoperable cancer at laparotomy. BSC cost varied considerably; a standard BSC pathway cost assumed home-based care with no admissions to hospital or hospice. QoL adjustment to generate QALYs is based on a heterogeneous data set, and it is likely that the QoL reduction in those undergoing perioperative chemotherapy would have been longer than in those undergoing surgery alone. Treatment costs were not assessed at a patient level and did not account for the heterogeneity in durations of hospital stay, chemotherapy and operation-related morbidity, which affects as many as 38 per cent of patients^5^. Despite these limitations, the present study has several strengths, benefiting from robust follow-up data with accurate causes and dates of death obtained from the Office of National Statistics with more than 75 per cent of patients followed for at least 5 years or until death. Patients were included consecutively from a single UK geographical region, and treated by the same multidisciplinary team, using a standardized treatment algorithm, with well audited and published quality control. Surgical outcomes were similar to national trial and audit data in terms of postoperative outcomes and cumulative survival^4^.

The three-fold increase in cost-effectiveness of surgical and oncological therapies strongly supports early diagnosis of gastric cancer, with attempted curative strategies as cost-effective.
